# Extra Limites

**Published:** 1822-03-01

**Authors:** 


### EXTRA-LIMITES.

### Medical Botany.

In our Review for September 1820, we had occasion to notice the Lectures given on this subject by Dr. Emerson, Member of the Royal College of Physicians, &c. &c. and laid before our readers a general outline of the Course. We are happy to find that the Doctor has continued his labours in this branch of Medical Education; and that he has considerably extended his course, which for the future will be confined to the summer months; thereby not interfering with the more important avocations of the student. We consider it totally superfluous on our part to enlarge on the great utility of this department of Natural History to the Profession, as it now very properly forms an *indispensable* qualification of its different members. This Series of Lectures embraces, agreeably to the schedule below, *Practical*, *Theoretical*, and *Medical Botany*; the last occupying the fourth and fifth division of the Course; which concludes with a history of the *Principal Medicinal Plants* employed by the native practitioners of India. The *general plan* of the Course we now beg to present to the public; it is as follows:—I. The first division has for its object—1st. The examination of the constituent parts of a *Flower* on which *Generic Character* is founded.—2d. The examination of the different parts of a *Plant* from which *Specific Characters* are taken.—3d. Nomenclature. II. The second division consists in the illustration of the *Linnæan* System. III. The third division is occupied by the consideration of the *Anatomy* and *Physiology* of Plants. IV. The fourth division comprehends *Vegetable Chemistry*. V. The fifth and last division includes the demonstration of the principal *Natural Orders of Jussieu*, and of the Medicinal Plants. In this section the *analogy* between the *forms* and *preparations* of Plants is explained agreeably to the latest discoveries of scientific Botanists. The detail of the above outline forms a part of the Introductory Lecture, which will be delivered in the first week of May at the Anatomical Theatre of Joshua Brookes, Esq. F. R. S. F. L. S. Soc. Cæs. Nat. Cur. Mosq. Soc. &c. &c. Blenheim Street, Great Marlborough Street. Occasional *examinations of the pupils* are held at the Lecturer's house in Sloane Street; where also they have access to an extensive Herbarium, and a select botanical library. In addition to these advantages, we understand Dr. E. has made arrangements for the introduction of the students into the principal botanical gardens in the vicinity of the metropolis; when they will be allowed to *dissect flowers* under his immediate inspection.

