# Erratum in Last No.

**Published:** 1822-06-01

**Authors:** 


*Erratum in last No.*

No. 8, p. 907, (Dr. Emerson's Prospectus,) for "forms and *preparations*," read "forms and *properties*," of vegetables.

